# Thoracic limb simulator for veterinary vascular access training

**DOI:** 10.1590/acb411226

**Published:** 2026-03-20

**Authors:** Ana Clara Nogueira Espinha, Julia Piolla, Patrick Carmona Marinho, Yuri Karaccas de Carvalho

**Affiliations:** 1 Universidade Federal Fluminense – Faculdade de Veterinária – Niterói (RJ), Brazil.

**Keywords:** Educational Technology, Education, Veterinary, Printing, Three-Dimensional

## Abstract

**Purpose:**

To develop and validate a thoracic limb simulator for veterinary vascular access training (TSVT), focusing vein puncture in dogs.

**Methods:**

The simulator was designed, three-dimensional printed, and filled with silicone and resin. Five experienced veterinarians, with no prior simulator experience, performed procedures including hand positioning, vein palpation, antisepsis, venipuncture, catheter insertion, and access maintenance. Evaluators completed an anonymous questionnaire with a 3-point scale and provided qualitative feedback.

**Results:**

The TSVT accurately reproduced thoracic limb and cephalic vein. All professionals reported appropriate hand positioning, vein palpation, and antisepsis. Blood collection was feasible for all evaluators, while catheter insertion was feasible for four and partially feasible for one due to resistance within the simulated vessel. Fixation and maintenance of vascular access were successfully performed by all. The simulator allowed repeated practice without live animals, supporting safe and controlled development of psychomotor skills.

**Conclusion:**

These results demonstrated that the TSVT is effective for training cephalic vein puncture in dogs, faithfully reproducing anatomical structures and enabling acquisition of clinical and psychomotor skills in a safe, ethical, and repeatable manner, representing a valuable tool for veterinary education.

## Introduction

Simulators can be defined as devices capable of replicating real patients, allowing students and professionals to practice and refine their anatomical, clinical, and psychomotor skills in a safe and efficient manner. The use of simulators in veterinary medical education has evolved significantly, being widely valued and implemented for enabling teaching without the need for live animals or cadavers. This alternative allows a more practical, standardized, and controlled approach to teaching, making the academic environment safer for both students and animals^1^.

The main advantage of using simulators lies in the absence of serious consequences in the event of an error, since by minimizing the use of animals, students and professionals have multiple opportunities for repetition without causing harm to the patient. Similarly, the models are available for use almost without limitation, requiring no time constraints for their execution. Moreover, students and professionals can develop the proposed skills without the pressure present in a real clinical environment, so that when they perform the practical activity, they do so with greater confidence and proficiency^1^.

Venipuncture is one of the most common procedures performed in a veterinary clinic, whether for collecting biological material or maintaining access to the patient. A well-performed venipuncture requires adequate and gentle control of the animal, correct location of anatomical landmarks and blood vessels, adequate restraint, and smooth, consistent movements of a needle or catheter to ensure the success of the procedure. Therefore, venipuncture should be regarded as a psychomotor skill to be acquired, with learning achieved through repetitive practice^2^.

The main vascular access used by professionals in dogs and cats is the cephalic vein. This vein is routinely employed due to its clinical versatility, given its superficial and accessible location, ease of blood flow occlusion (tourniquet), and a course with few bends and adequate diameter^3^.

Considering the above, simulating vascular access, particularly of the cephalic vein, becomes essential for comprehensive professional training. The present study aimed to develop and validate a thoracic limb simulator for veterinary vascular access training (TSVT).

## Methods

The study was conducted at the Laboratory of Veterinary Educational Technologies, located in the Faculty of Veterinary Medicine at the Universidade Federal Fluminense, in Niterói, Rio de Janeiro, Brazil. The creation of the simulator did not involve the use of live animals or cadavers and, therefore, was exempt from registration with the Ethics Committee on the Use of Animals.

**Figure 1:**
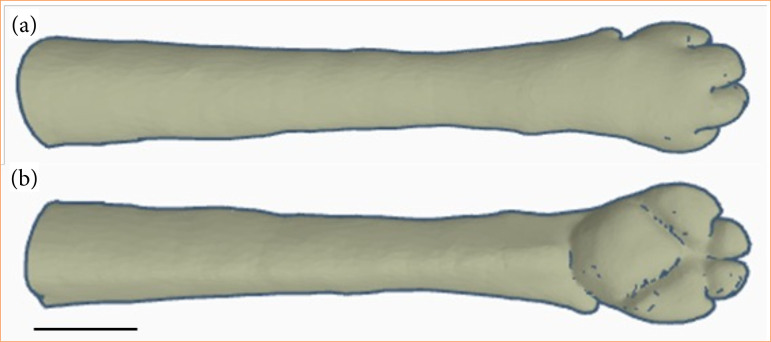
Digitalization of the middle and distal third of a dog’s thoracic limb. (a) Cranial and dorsal view. (b) Caudal and palmar view.

Source: Elaborated by the authors. Scale bar: 2 cm.

Initially, a literature review was conducted on the anatomy (skin, musculature, bones, and blood vessels) of the canine thoracic limb. The design of the simulator’s external conformation was performed using Thingverse (Autodesk, Inc., San Francisco, California, United States of America) and ZBrush (Maxon Computer GmbH, Bad Homburg, Germany) software, aiming to reproduce the external anatomical details of the canine thoracic limb (Fig. 1). Subsequently, the simulator was manufactured using a three-dimensional (3D) printer model A2V2 (GTMax3D, Americana, São Paulo, Brazil) with acrylonitrile-butadiene-styrene (ABS) filament.

Subsequently, the 3D model of a dog’s thoracic limb received a light layer of resin to remove any defects that may have occurred during printing. Then, the 3D limb received three layers of silicone. The first layer covered the printed structure. The second was applied along with the flexible tube that mimicked the blood vessel, and the third covered the previous layers, completing the simulator. The dimensions of the TSVT produced were: 180 mm (length), 20 mm (width), and 25 mm (height).

The simulation of the cephalic vein was performed using a flexible silicone tube with the following dimensions: 4 mm in diameter, 1-mm wall thickness, and a hollow section mimicking the vein lumen (2 mm). Blood was reproduced using red food coloring.

These steps aimed to replicate the tactile anatomical properties, mainly of the middle third of the forearm, the region most used for vascular access. In this region, we were able to mimic the skin and the extensor carpi radialis and flexor carpi radialis muscles (silicone layers). Furthermore, the flexible tube was positioned craniomedial to the printed prototype, respecting the path of the blood vessel in the middle third of the forearm. In a deeper layer, the 3D printed part reproduced the rigid (bony) portion of the forearm.

The sharp materials used in the procedures, such as hypodermic needles, scalpels, and intravenous catheters, were of a diameter compatible with that of the simulated vessel.

The simulator was tested by five experienced veterinarians in vascular access on live animals, who had never previously used simulators for this type of training. In this study, the simulator was tested by professionals, since they had mastered the procedures and, therefore, could thoroughly evaluate the reliability of the TSVT for learning these clinical skills.

The professionals evaluated the reproduction of the anatomical details of the thoracic limb, including aspects related to hand positioning by the operator and palpation of the simulated cephalic vein. The evaluators also assessed the feasibility of the following procedures: antisepsis of the vascular puncture site; blood collection using a hypodermic needle and scalp; intravenous catheter placement and fixation; and maintenance of intravascular access.

Thereafter, the professionals completed an anonymous questionnaire containing questions rated on a 3-point scale (1 = feasible, 2 = partially feasible, 3 = not feasible), in addition to a section for describing their impressions, which could justify the score assigned to the simulator during its handling.

## Results

The thoracic limb simulator for veterinary vascular access accurately reproduced the anatomical structures necessary for performing the main routine procedures via vascular access. Furthermore, the proportions of dimensions such as diameter, width, and thickness inherent to the thoracic limb of a medium-sized dog were preserved. The cephalic vein, the main access vessel in the canine thoracic limb, was replicated in the TSVT. Among the intrinsic characteristics that were reproduced are its superficial location, cranial projection, and a course with minimal curvature along the forearm region.

**Figure 2:**
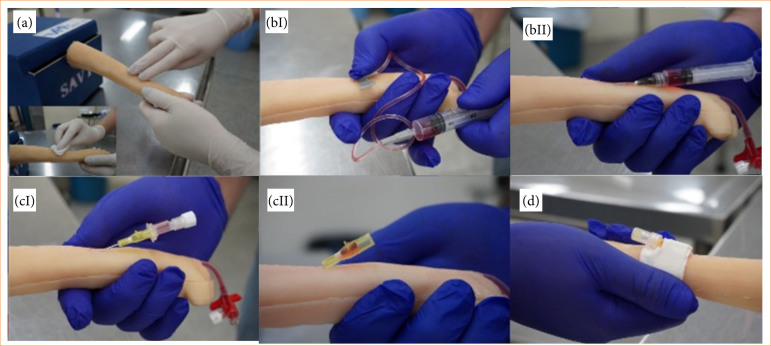
Procedures performed on the veterinary vascular training simulator. (a) Hand positioning, venous palpation, and antisepsis of the region. (b) Blood collection using sharp materials (scalpel and hypodermic needle, respectively). (c) Peripheral venous catheter placement. Note the presence of artificial blood in (cI) the puncture chamber and in (cII) the connection cone. (d) Fixation and maintenance of vascular access.

Source: Elaborated by the authors.

The TSVT allowed hand positioning similar to that used on a dog. Palpation of the simulated cephalic vein was possible along its entire course by all professionals (Fig. 2a and Table 1). Furthermore, the simulation of antisepsis training at the site was evaluated and considered satisfactory by all evaluators. In these non-invasive aspects, 100% of the evaluators deemed the simulator suitable for training.

**Table 1:** Percentage of procedure execution levels performed by evaluating professionals on the veterinary vascular training simulator.

Procedure/concept	Feasible	Partially feasible	Not feasible
Hand positioning and palpation	5	0	0
Blood collection	5	0	0
Catheter placement	4	1	0
Fixation and maintenance of vascular access	5	0	0

Source: Elaborated by the authors.

Blood collection using sharp materials, such as hypodermic needles and scalpels, on the TSVT was considered feasible by all evaluating professionals. The evaluators reported being able to access the vascular bed along its entire course as replicated in the simulator. Furthermore, they described the simulation of this procedure as comparable to and easily performed in previous experiences with live animals prior to the study (Fig. 2b and Table 1).

The third aspect evaluated was the placement of an intravenous catheter. Four of the five professionals considered the procedure feasible. One evaluator described the procedure as partially feasible. The positioning of the catheter needle within the lumen of the simulated vessel, as well as the administration of substances, was reported as easily performed by all evaluators. However, the professional who rated the procedure as partially feasible reported some resistance when advancing the catheter within the simulated vessel and needed to repeat the procedure, replacing the catheter (Fig. 2c and Table 1).

The procedures for securing and maintaining intravascular access were considered feasible by all evaluators. They reported adequate adhesion of the adhesive tape during fixation of the catheter and the PRN adapter to the TSVT (Fig. 2d and Table 1).

## Discussion

Vascular procedures are indispensable in veterinary medical practice. Venipuncture and catheterization are considered techniques that often cause insecurity among professionals, especially among veterinary medicine undergraduates. The use of simulators has proven to be an effective means of ensuring the learning of these and other procedures, while simultaneously fostering the self-confidence of their practitioners^4^.

The introduction of simulators during veterinary medical training enables students to practice the proposed clinical skills increasingly early in their curriculum. It also allows these techniques to be revisited throughout the undergraduate program, ensuring long-term skill development^5^.

The use of simulators during training reduces the need for live animals in teaching practices, aligning with the principles of the three Rs (replacement, reduction, refinement) and animal welfare^6,7^. In this context, the use of the TSVT for training veterinary students and professionals adheres to these principles by eliminating the need for both live animals and cadavers.

The choice to have the TSVT evaluated by experienced professionals was necessary, as their prior knowledge would indicate whether the proposed procedures could be performed. The results obtained using the TSVT could then be compared with the evaluators’ previous experiences handling live animals^8^.

A study conducted at the Lincoln Memorial University College of Veterinary Medicine developed a simulator similar to TSTV and reported the same findings regarding the training of these skills. Before introducing the venipuncture model to students, the authors conducted an evaluation with specialized professionals to determine whether the simulator validly represents reality^9^.

Regarding anatomical and functional correlation, the TSVT successfully reproduced anatomical structures such as skin, radial carpal extensor and radial carpal flexor muscles, the middle third of the forearm, and the cephalic vein. These structures are inherent to the canine thoracic limb and important for the full execution of the proposed procedures. These positive results demonstrated that the design, construction, and materials selected for the creation of the TSVT were effective. Similar results were reported in a study that developed a 3D model of a canine skull for anatomical learning, in which it was observed that the precise combination of design and execution in the creation of the simulator ensures quality and functionality^10^.

Additionally, one of the evaluators reported slight difficulty when advancing the catheter within the simulated vascular bed. We believe this difficulty may have been caused by friction between two abrasive materials: on one side, the material composing the catheter, and on the other one, the silicone forming the simulator. The study that took place at Lincoln Memorial described similar reports from students and evaluators in their feline venipuncture model, and further stated that they did not find a material capable of perfectly replicating an animal’s blood vessel^9^.

Some of the main complications arising from peripheral venipuncture in dogs are vessel rupture, bleeding, and hematoma. In this sense, studies have indicated that, after training with low-cost simulators, there was a reduction in the number of animals experiencing complications following puncture. These findings corroborate the present study, since similarly, the use of the TSVT may also contribute to mitigating such complications when used in the acquisition of clinical skills by future professionals^11,12^.

All professionals considered the TSVT suitable for non-invasive procedures, such as venous palpation, hand positioning, and antisepsis simulation. These results highlight the relevance of these fundamental steps for vascular access, indicating that their training is indispensable to reduce errors and increase safety during real procedures.

Antisepsis, for example, is an essential step in both venipuncture and intravenous catheterization. A study conducted at the University of Tennessee demonstrated knowledge retention following the use of a canine mannequin model in practical classes. The researchers noted that although many students successfully performed blood collection or catheter placement, they forgot some steps, such as antisepsis, resulting in lower scores^12^. Therefore, the possibility of performing all steps related to vascular access and maintenance in our study reinforces the idea of assisting in step-by-step consolidation of these procedures.

Moreover, although the central objective of the study was not the application of the TSVT in practical assessments, this simulator could help measure the actual learning of these procedures by future professionals without the need to use live dogs. As next steps, considering that the TSVT has demonstrated potential for replicating the learning of these clinical skills, we intend to apply it to evaluate the degree of learning and satisfaction of students when using the simulator in their process of acquiring these clinical skills.

On the other hand, the methodology used for the design and construction of the TSVT can serve as a basis for the creation of other veterinary training and learning simulators, such as models of dogs of different sizes or other species.

## Conclusion

The TSVT proved to be effective for practical training in vascular access, encompassing preparatory aspects, as well as invasive procedures and access maintenance.

## Data Availability

Data sharing is not applicable.
